# Patients' perceptions of quality in Swedish primary care – a study of differences between private and public ownership

**DOI:** 10.1108/JHOM-09-2020-0357

**Published:** 2021-03-22

**Authors:** Thomas Andersson, Nomie Eriksson, Tomas Müllern

**Affiliations:** School of Business , University of Skövde , Skövde, Sweden; School of Technology and Society , Skövde, Sweden; Jönköping International Business School , Jönköping, Sweden

**Keywords:** Perceived quality, Primary care, Ownership

## Abstract

**Purpose:**

The purpose of the paper is to describe and analyze differences in patients' quality perceptions of private and public primary care centers in Sweden.

**Design/methodology/approach:**

The article explores the differences in quality perceptions between patients of public and private primary care centers based on data from a large patient survey in Sweden. The survey covers seven dimensions, and in this paper the measure Overall impression was used for the comparison. With more than 80,000 valid responses, the survey covers all primary care centers in Sweden which allowed for a detailed analysis of differences in quality perceptions among patients from the different categories of owners.

**Findings:**

The article contributes with a detailed description of different types of private owners: not-for-profit and for profit, as well as corporate groups and independent care centers. The results show a higher quality perception for independent centers compared to both public and corporate groups.

**Research limitations/implications:**

The small number of not-for-profit centers (21 out of 1,117 centers) does not allow for clear conclusions for this group. The results, however, indicate an even higher patient quality perception for not-for-profit centers. The study focus on describing differences in quality perceptions between the owner categories. Future research can contribute with explanations to why independent care centers receive higher patient satisfaction.

**Social implications:**

The results from the study have policy implications both in a Swedish as well as international perspective. The differentiation between different types of private owners made in this paper opens up for interesting discussions on privatization of healthcare and how it affects patient satisfaction.

**Originality/value:**

The main contribution of the paper is the detailed comparison of different categories of private owners and the public owners.

## Introduction

Many countries have healthcare systems that are open for both public and private care providers ( Sheaff *et al.* , 2019 ). Several countries have experimented with deregulation, privatization, customer-choice models and other ways of introducing private providers into the healthcare system. Sweden has been in the forefront of this wave of experiments, and has gradually transformed a system of primarily publicly owned providers to a mixed model with private providers working alongside public providers ( Maun *et al.* , 2013 ). The changes have been most radical in primary care, where all regions in Sweden have implemented a model where patients can choose a primary care center from a list of both private and public providers ( Isaksson *et al.* , 2016 ; Vengberg *et al.* , 2019 ). It is also important to mention that, in Sweden, both public and private providers of primary care are funded through taxes. This raises interesting questions of whether quality has increased in the whole system, and whether there are differences between private and public primary care centers. The extant literature is not entirely conclusive, although a number of studies in a Swedish context show that private providers typically have higher quality perceptions ( Glenngård, 2012 ; Maun *et al.* , 2015 ). More data-driven analyses of patients' quality perceptions of private and public providers of primary care are clearly needed.

This paper addresses whether there are differences in patient's quality perceptions of private versus public providers of primary care. Previous research has consistently treated private providers as one, homogenous, group ( Maun *et al.* , 2015 ), thereby neglecting the variation between different categories of private owners. It has been argued that the distinction between for-profit and not-for-profit providers is important, and a few studies have provided results comparing the two types ( Comondore *et al.* , 2009 ; Eggleston *et al.* , 2008 ; Kraska *et al.* , 2016 ). In this paper we argue that further distinctions can be made, for instance between larger corporate groups and smaller, independent actors.

The study was done in the context of the nationwide reform in healthcare in Sweden. In 2010, primary care throughout Sweden was opened for private providers; to date, around 42% of the centers are privately owned (in 2017 there were in total 1,150 centers). This provided a good arena for a nation-wide comparison of perceived quality of care between different owner categories. Starting from a low level in 2010 (and some years before that in selected regions) the number of private providers has rapidly grown and is expected to reach 50% of the total number within a few years. The private providers are publicly funded (through the region where they operate) based on the number of patients listed at the single center (adjusted for a number of parameters, such as type of patients, socio-economic factors, and areas of specialization), and, in some cases, based on the actual amount of medical treatments and consultations.

The purpose of the paper is to describe and analyze differences in patients' quality perceptions of private and public primary care centers in Sweden. It further compares the quality perceptions for different categories of private centers.

## Literature review

The research on patients' quality perceptions in healthcare show mixed results when comparing private providers and public ones. Some studies support the view that public providers have higher patients' quality perceptions ( Jabnoun and Chaker, 2003 ), whereas others show the opposite result ( Polsa *et al.* , 2011 ; Shabir *et al.* , 2016 ). There are also studies that show ambiguous results concerning ownership and patients' quality perceptions ( Angelopoulou *et al.* , 1998 ), It is important to acknowledge that the extant literature rarely focuses on primary care, with most studies comparing quality perceptions for different owners of hospitals ( Eggleston *et al.* , 2008 ; Owosu Kwateng *et al.* , 2019 ; Tengilimoglu *et al.* , 1999 ) and healthcare as a whole ( Owusu-Frimpong *et al.* , 2010 ). In the Swedish context, there is research ( Glenngård, 2012 ; Maun *et al.* , 2015 ) supporting the overall observation that private primary care centers have a higher patient quality perception than public centers, but little is known about the more detailed patterns.

In parallel with studies on quality perceptions, there are studies that focus on patient satisfaction. Although patient satisfaction is a different (although related) construct than quality perception ( Bitner and Hubbert, 1994 ; Johnston, 1995 ; Taylor and Baker, 1994 ), it still sheds light on the effects of introducing private providers. Burström *et al.* (2017) report that patient satisfaction for primary care as a whole was unchanged after the reform. Their study, however, did not differentiate between different ownership categories. Some research shows lower patient satisfaction for private, for-profit – as compared to private not-for-profit – hospitals, at the same time as public hospitals yield lower satisfaction than their not-for-profit private counterparts ( Kraska *et al.* , 2016 ). Furthermore, no research has studied the differences within the broad group of private providers of primary care. Private primary care centers are usually treated as one group, and this does not reflect the variation within the broad group of private centers. This article provides a detailed analysis of different categories of private owners.

International studies on hospitals and healthcare in general show mixed results, and we agree with Eggleston *et al.* (2008) that comparisons between countries are difficult due to differences in institutional structures and different ways of conceptualizing quality in healthcare. In a meta-review of studies on quality of care in nursing homes, Comondore *et al.* (2009) showed a higher quality (as measured by a combination of input, process and outcome measures) for not-for-profit nursing homes. The study did not differentiate between public not-for-profit and private not-for-profit. Kumaraswamy (2012) provided empirical results on healthcare centers in India, showing more positive patients' quality perceptions for corporate compared to non-corporate (the study was not clear on whether non-corporate is equal to public) centers. Barhem *et al.* (2010) have added important insights on differences between for-profit and not-for-profit health care organizations, in their case, based on employee satisfaction in the two ownership categories.

In parallel with the discussion on quality differences between private and public ownership, there has also been a fierce political discussion in Sweden, with a criticism of private actors being involved in healthcare. The discussion has focused on a number of issues, such as big company groups, and what is perceived as an unjust earning of profits in the healthcare sector, as well as an uneven utilization of healthcare in the population ( Beckman and Anell, 2013 ; Isaksson *et al.* , 2016 ; Burström *et al.* , 2017 ). Less is known, however, about patients' perception and attitudes towards private primary care units, and to what extent perceptions and attitudes are formed based on the broader political discussion or on the actual care and service patients receive. It is important to acknowledge that the general attitude towards privatization is a different construct than quality perceptions. It is possible that a person can have a negative view on privatization in general, and still have a positive quality perception when given care from a private provider. Sweden being at the forefront of mixing private and public ownership in healthcare, we argue that the results from this study are germane for countries that are aiming at similar changes in their healthcare systems.

The paper analyzes data from a survey of patients in Swedish primary care (National Patient Survey), that measured their perceptions of the care they received. Selected patients had visited a primary care center and met a physician in the year of the survey. Patient quality perception is here defined as a process through which the patients' expectations are balanced with the perception of the care they receive ( Alrubaiee and Alkaa'ida, 2011 ; Newsome and Wright, 1999 ). This definition is in line with the broader definition of service quality offered by the service management literature (see, for instance, Grönroos, 1990 ). It is generally acknowledged in the literature on service management/marketing that services are fundamentally different from goods, and that the production of services relies on a different logic. Patients nowadays are arguably more informed and active in seeking the best possible care, and this calls for a redefinition of their role and importance as a part of the healthcare system ( Liff and Andersson, 2011 ). Joiner and Lusch (2016) make a case for introducing a Service-dominant (S-D) logic in healthcare, stressing the co-creation between patient and provider. In the survey, the patient is asked several questions on how they perceived different aspects of the last visit they made to a primary care center. Many authors argue that learning about the patients' perceptions of the services they receive is an important basis for improving the quality of healthcare ( Senic and Marinkovic, 2012 ), and that a more patient-centered approach is called for when competition between providers is increasing ( Pihlainen *et al.* , 2019 ).

The paper contributes to a growing stream of research on perceived quality in healthcare in general ( Joiner and Lusch, 2016 ; Senic and Marinkovic, 2012 ), and differences between different types of ownership in particular. Although it is methodologically hard to compare research between countries due to lack of data, and also due to differences in healthcare systems, we still argue that the analysis of different categories of private owners is relevant beyond a Swedish context.

## Methodology

In the empirical part of the paper, some basic demographic data on the population of primary care centers are provided and results from the quality survey are presented and analyzed. With the introduction of private providers of primary care in the early 2000 (and fully implemented by 2010), the public ownership was replaced with a mixed model, with some degree of competing for patients, not only between private and public providers, but also within the group of public providers. An important aspect of the reform was to create neutral conditions for both public and private providers, which meant that the publicly owned primary care centers were expected to operate under the same conditions as privately owned. The introduction of private providers of healthcare was driven by several political arguments by the advocates of private care. Besides the financial argument that private providers would increase productivity, the reform was motivated in terms of increased freedom of choice for patients ( Maun *et al.* , 2013 ). It was argued that allowing patients to freely choose the primary care center would put pressure on the providers to give better services, and this would increase quality in the whole system.

At the beginning of 2017, there were a total of 1,150 primary care centers in Sweden, with 42% being private and the remaining 58% being public. The number of public centers used in the study is 662. The group of private care centers is diverse; for the purpose of this article, they were divided into four different groups, totaling 455 centers:

*Independent units, for profit (Ifp)* : This category consists of small companies operating up to four primary care centers that do *not* have a clear not-for-profit profile. When the market for primary care was opened for private actors, starting gradually in a few regions already before 2010, this created opportunities for new actors to emerge in the healthcare sector. The system with public funding made it possible for completely new actors to start a primary care center. 155 out of 455 private primary care centers used in the study fall into this category.

*Independent units, not for profit (Infp)* : This category consists of actors that own up to four primary care centers, and that operate with an explicit not-for-profit profile. It includes a variety of different types of ownership and profiles, such as idea-based organizations (for instance with a religious base) and cooperatives (personnel cooperatives or member-owned organizations). In this category we find 15 centers.

*Company group, for profit (Gfp)* : This category consists of companies that own five or more centers. In this group we find several companies that already existed in the market, for instance operating elderly care, schools, social care and similar welfare tasks. These companies could, with the new legislation, expand into primary care. This category is the largest of the private groups and consists of 279 centers.

*Company group, not for profit (Gnfp)* : This category has a similar profile as Infp, but owns five or more units. This category consists of 6 centers.

The distinction between the categories is not always entirely clear, with some actors having traits of two categories. A good example is the big group Praktikertjänst that can be described as a producer-cooperative, but at the same time operates with a clear for-profit mission and has the legal structure of a limited company. To be consistent, we have used the presence/absence of a profit mission as the distinguishing mark between the categories, in this case, classifying Praktikertjänst as a Company group, for profit.

The survey covers different dimensions of the perceived quality (overall impression, emotional support, respect and kindness, availability, participation and involvement, continuity and coordination, information and knowledge) that a sample of patients from each of the primary care centers that visited a physician during a specific month (September) fills in. The population consists of all patients that visited a physician in primary care during this month; from this, a random sample is drawn, and the survey is sent in digital form to the selected patients. The sample drawn from each primary care center is based on and reflects the total number of visits made to that center during the selected month. Four waves of survey results were available for analysis (2011, 2013, 2015 and 2017; data for 2019 were not yet available when the article was completed). For the 2017 survey, 80,000 surveys were completed. The data can be broken down to the level of the single primary care center, making it possible to single out the results for the private and public care centers.

For this article, it was decided to present the results for the measure Overall Impression as the basis for comparing the different owner categories. This measure asks the patient to summarize the perception of the primary care unit, and therefore provided a strong overall indication of how the patients evaluate the care they received. The remaining six measures pick up on different aspects of the patients' evaluation of the care center. A detailed presentation of all seven measures in the survey was judged to be beyond the scope of this article. This choice was motivated along the following lines. Although service quality arguably is built on different service determinants ( Johnston, 1995 ), it is often argued in the service marketing literature that selecting a key ratio and/or measure can be used to monitor the degree to which a company is retaining its customers ( Hu *et al.* , 2009 ). The use of Overall Impression has been used in numerous studies of health care ( Attree, 2001 ; Gray and Boshoff, 2004 ), and we furthermore draw on the argument that patients form a strong first impression of the service provider ( Rimondini *et al.* , 2018 ). Overall Impression asks the customer (patient) to make an overall evaluation of the quality, and can therefore be used as an indication of the perceived service quality. The main point of this article is not to make a detailed study of the determinants of patient perceptions of service quality; rather it is to compare the different categories of owners. Based on the arguments above, it was deemed sufficient to use the summary measure of overall impression.

For this article, it was decided to include all primary care centers that had valid answers on Overall Impression in the two latest surveys (2015 and 2017). Care centers with answers in only one of the two surveys were therefore excluded. The main argument for this was to allow for a check of how consistent the results were over time. After this procedure, 1,117 primary care centers remained and were used in the analysis. The empirical results are presented and discussed in three steps. In the first step, the overall results for the measure Overall Impression are presented for the two groups, private and public primary care centers in each of the 21 regions. In the second step, the private providers are more carefully examined to see whether there are any differences between the four categories of private owners, for each of the regions. In the third step, the four categories of private owners are compared with the public owners, and the differences between the owner categories are also aggregated for Sweden as a whole. Is the quality survey with completed answers from 1,117 out of 1,150 primary care centers a relevant (valid) and reliable source to reflect upon the quality of primary care in Sweden? With 80,000 responses, it is arguably one of the largest surveys conducted in Sweden; furthermore, it is done every second year, which makes it possible to create time-series. The survey instrument is freely available, and the results are open for researchers, which makes it easy to check how the survey is constructed and the methodology.

## Empirical results

### Overall comparison of quality perceptions for private and public providers

In the table below, we summarize the results for 2015 and 2017 on the measure Overall Impression, for all primary care centers in each the 21 regions of Sweden. The table was created in several steps that are explained below:

Step 1: After ordering the primary care centers from highest to lowest value on Overall Impression in each of the 21 regions, we then identified the median in each region. The median for each region was calculated as the value for the primary care center that was in the middle of the list within each of the 21 regions. The analysis is done for 2017, but figures for 2015 are also included to show changes over time. We are consistently interested in how each owner category scores relative to the other category, and it was a deliberate choice to not analyze the actual values. Based on this, using the median was sufficient. Deeper descriptive statistical analysis would not add any details to the relative comparison of the owner categories. The research group has done detailed statistical analysis of the data for other purposes and that did not reveal any change from the results presented here.

Step 2: For each region, we next counted the percentage of public providers with valid answers that had a score over the median for Overall Impression (see step 1 above) as a percentage of the total number of public providers in each region. (See columns 2 and 3 in Table 1 , and columns 4 and 5 for the private providers). The same procedure was done for the private care centers.

For the first region, Blekinge, six public care centers of 13 total, or 46%, had a score over the median; the score for the private ones was 67% (four out of six above the median). In this way, we have created a measure of how well the public and providers are represented (in relative terms) on the upper half of each measure. To show the consistency of the figures, we have also added the figures for the survey done in 2015 in brackets after the figures for 2017.

The table shows clearly that quality perceptions for private providers are high (as measured by Overall Impression), with a larger proportion of care centers above the median compared to public centers in 17 of the 21 regions (the same number in 2015). For the remaining four regions, private providers have a lower percentage than public ones (in 2015, three regions had lower scores and in one region the score was equal).

Comparing the changes between 2015 and 2017, some observations can be made. For the public providers, seven regions had a better result for 2017 compared to 2015, while in two regions the percentage was lower; in 12 regions the score was unchanged. For the private providers, the pattern was similar, with six regions having a better score for 2017. In five regions they had a lower score, while for 10 regions the score was unchanged. For single regions, there are, in some cases, big changes – for instance, for Blekinge and Jönköping – but the overall pattern is clear: the privately owned primary care units have consistently higher quality perceptions as measured by Overall Impression. Despite individual variations in some regions, the data consistently point in the same direction: the private primary care centers have higher quality perceptions than the public ones.

### A detailed analysis of differences between the four categories of private owners in each region

The next step was to make a more detailed analysis of the private providers. As presented above, we have split the private primary care centers in four different ownership categories. This required gathering information on ownership for each single care center. Since there is no central register, this had to be done manually, center by center, using various public sources. The database created for this purpose thus contains information that is not available anywhere else. In this part of the empirical results, we were interested in differences between the four categories of private owners. Was there a pattern in terms of which category of private owners yielded the highest quality perceptions?

In the table below, we have analyzed the 455 private care centers that had valid results in both 2015 and 2017 and created one column for the ones with scores over the median for each region, and one for the centers with scores below the median (see step 1 above for a description of how the median was used). For each column and for each region (each cell in the table), we calculated the percentage of the centers falling in each of the four categories of private owners. For the first cell, that is, private providers over the median for Blekinge, there was a total number of 4 ( *n* = 4; see step 2 above for an explanation of how this number was identified), with one (25%) being independent for profit (Ifp), one (25%) being independent not for profit (Infp) and two (50%) belonging to large corporate groups, for profit (Gfp). At the bottom of the table, the figures are summarized for Sweden as a whole.

Several observations can be made based on this table. Perhaps most striking is the low number of not-for-profit actors in the sample. We find not-for-profit actors (Gnfp and Infp) in only eight of the 21 regions, and, in all cases, in small numbers compared to the for-profit actors. In total, there are 21 not-for-profit primary care centers in the sample of 455 private centers. Furthermore, the majority of the not-for-profit actors in the sample fall in the category Independent not-for-profit (15 centers), with only one actor (operating in two regions) representing the Corporate group, not-for-profit (in total, they own six centers). These are surprising results considering the fairly low barriers of entry that would not favor any particular owner category. We would expect primary care to be an attractive and viable arena for not-for-profit actors. The independent not-for-profit centers get good scores in the survey, with 13 out of the 15 Infp falling above the median, although the scores should be interpreted carefully due to the small number of not-for-profit centers in the sample.

The large corporate groups (for-profit) dominate the sample, with 279 of the 455 private care centers (61%), and with Independent (for-profit) being the second-largest private owner category (with 155 centers, 34%). The large corporate group (for profit) also has the largest share among the private owners above the median, with 54.7% of the sample (152 out of 278). The Independent for-profit (Ifp) group accounts for 39% of the private centers above the median. The large corporate groups have a smaller share of the private centers above the median compared to their share of the total number of private centers. The Independent for-profit group, in turn, has a larger share of the private centers above the median compared to their share of the total number of private centers. This clearly indicate a higher patient quality perception for small, independent actors compared to the corporate groups.

Looking below the median, we see a similar pattern with higher quality perceptions for the small, independent actors compared to the corporate groups. The Independent for-profit (Ifp) group accounts for 26% (46 out of 177) of total number of private centers below the medium (considerably lower than their 34% share of the total number of private centers). The Corporate groups (Gfp) account for 71.8% (127 out of 177) of the total number of private centers below the medium, which is considerably higher than their 61% share of the total number of private centers.

### A detailed analysis of differences between public and the different types of private owners

In the final step of the data analysis, we deepen the analysis of the different owner categories. We use the same data as presented in table two above, but this time we add the public ones. As before we do the analysis for each region and then summarize the results for Sweden as a whole. Compared to table two the new table below shows the result per owner category to facilitate the comparison between the five owner categories. In the table below, each row counts the number of centers, in each owner category, falling above and below the median for that region. At the bottom of the table the results are aggregated for Sweden as whole, to allow for a comparison between the five owner categories, in terms of how well they are represented on the upper half in the sample for each owner category. In the first column, Independent for-profit we see that 109 out of 155 are place above the median in their region and 46 below the median. For this category around 70% of the total number of centers are placed above the median.

The table above confirms the observations from the previous two steps, and it adds a more detailed comparison between the different owner categories (this time including the public providers). The comparison of the number of centers above and below the median within each owner category allows for a pedagogical evaluation of the Overall Impression measure. What is striking are the low numbers for the public providers, with more public centers with a score below the median than above (only 43.8% are above the median in their region). Consistent with Table 2 , we also see that the large corporate groups (Gfp) have a lower score compared to the independent centers (152 out of 279 above, and 127 out of 279 below the median). The 54% of the Gfp centers above the median can be compared with the 70% for the Independent for-profit (Ifp) centers. For these three owner groups, the number of centers in each category is high, which makes the figures robust for Sweden as a whole. It can be argued that an equal number of centers above and below the median (which is the case for Gfp and the public ones) indicates that the scores are fairly evenly distributed from lowest to highest score, and, in that sense, the figures for Gfp and public centers are according to expectation. What stands out are the figures for Ifp (70% above the median), being too high to be evenly distributed. It is clearly so that small independent players (for-profit) are unique in enjoying a very high patient quality perception. The high figures for the Independent, not-for-profit (Infp) group is interesting, but the low number of units (15) calls for some caution in drawing conclusions from it.

The comparison was created based on the median for each of the 21 regions, which means that there was no absolute median for Sweden as a whole in the table. In order to check how robust the figures were, we also made a calculation without dividing the care centers per region and instead ranked them for Sweden as a whole. The results from this recalculation confirm the overall picture with some notable differences. For the Ifp, the new figures were slightly higher than above, and Gnfp was lower. For the Gfp, the figures were slightly lower and for the public they were nearly unchanged. For the remaining category, Infp, the figures changed caused by one center falling above instead of below the median, once again showing that the calculations are sensitive when there are small numbers of centers.

## Discussion

The analysis of quality perceptions for private and public primary care centers in Sweden has shown that private providers are overrepresented above the median for the measure Overall Impression in most regions. In that respect, the results from this study confirm the results from previous studies of Swedish primary care, showing that private providers have higher patient perceptions of quality ( Glenngård, 2012 ; Maun *et al.* , 2015 ). Adding to previous studies, the findings from this study show interesting differences between the different categories of private owners, and especially between the Independent for-profit (Ifp) and Corporate groups (Gfp). Through the comparative analysis in Table 3 , we showed a high quality perception for the independent units (with 70% of the Independent for-profit being above the median, compared to 53.8% for the corporate groups). Although the number of units in the not-for-profit category is small, it clearly shows a very high quality perception for the independent units compared to large groups and public ownership.

International research on differences between private and public ownership is, with some exceptions, showing a higher patient quality perception for private owners. Our literature review shows that most studies give support to a higher quality perception for the private providers ( Owusu-Frimpong *et al.* , 2010 ; Tengilimoglu *et al.* , 1999 ). The service marketing literature suggests that the quality of a given service is measured as the relation between the expectations on the service delivery and the perception of the actual outcome ( Grönroos, 1990 ). A high score in the patient survey used in this article indicates that expectations have been met or, more probably, exceeded. A low score can mean that the patient had low expectations and they were met, or, more probably, that the expectations were not met, generating a low score. The survey does not specifically measure the gap between expectation and service perception, but it is highly probable that the private, independent centers meet or exceed expectations to a larger degree than their public and corporate group counterparts. The survey does not replace the need to have a variety of measures of quality in healthcare, for instance, focusing more on actual outcomes (as reported in the growing number of national quality registers in Sweden). The survey is specifically focused on the patient's perception of quality and our focus is on how this differs between the owner categories. In that sense, we do not offer a general answer on what is quality in healthcare. Future research can shed more light on whether patient quality perceptions correlate with other outcomes (such as more consistent and evidence-based practices, patient safety, accidents, and other measures of the actual care given).

Neither the Swedish literature on patient quality perceptions in primary care, nor the international healthcare literature, has made a distinction between different categories of private owners. Through analyzing the different categories of private owners, this paper offers a detailed description that shows a considerably higher perceived quality for private, independent primary care centers. The access to data from all primary care centers in Sweden has given a unique opportunity to analyze the effects of ownership on patients' quality perceptions. An international comparison with the Swedish data is, for obvious reasons, hard to make, but there is no major reason to assume that the private independent groups are less popular than private groups in other counties.

It is beyond the scope of this article to give reliable explanations for these results, but some observations can be made. Some authors have argued, based on limited empirical data, that there is a selection bias in the sense that people from socio-economically wealthy areas are more prone to choose a private center ( Burström *et al.* , 2017 ). Does this automatically transfer over to a positive perception of the care they are given at the center? The data for the Independent for-profit (Ifp) centers seem to confirm this. On the other hand, this does not explain why the Group for-profit (Gfp) category has a substantially lower score. Future research can possibly verify whether there is such a socio-economic bias in the data, but it is beyond the scope of this article to show such patterns. Is it possible that the private centers have an incentive to influence the sample to include patients that are positive? They could, but they have little control over the sampling process, as it is carried out by an independent research company.

The data confirm that the independence of the single center is an important factor, possibly creating a perception of being small and close to the patient. It does not necessarily mean that the single center is small in terms of number of listed patients; in fact, some of the most popular independent centers have a large number of listed patients. Another potential explanation could be that patients are affected by the broader, ideological discussion on making a profit in healthcare. It is primarily the large corporate groups that have been criticized for making a profit, and there could be a guilt-by-association effect that influences the patients' perception of them.

A further explanation for the patient quality perceptions in the independent centers could be that the ownership of the center is closer to the operative management. In many of the centers, the owner is working at the center, and this could be an incentive to try to make sure that patients are satisfied. This is, however, hard to verify with the data from the survey, but it is at least consistent with the results, showing higher quality perceptions with independent centers compared to centers belonging to a larger group. Agency theory suggests that managers of private health care centers try to maximize their own utility, and thus have an incentive to run the unit in an efficient way ( Tiemann *et al.* , 2012 ). It can be argued that a center that is run in an efficient way probably yields a higher patient quality perception. This, however, does not explain the difference between the independent and corporate groups in our material.

This research only focused on the measure Overall Impression, which summarizes patient satisfaction with the care received. In future research, it is interesting to crosscheck the results for Overall Impression with the other six measures in the national survey, to see whether there are further patterns when comparing the owner categories. Future research can also analyze whether there are socio-economic explanations for the popularity of the private, independent care centers. This would require cross-checking the address for each care center, with data on income levels for the area. This is not possible without a lot of manual work. Addresses for the care centers, for instance, are not easily accessible for research purposes, but need to be collected region by region.

## Conclusions

This paper aimed at showing how patient quality perceptions differ between different ownership categories in Swedish primary care. The paper analyzed data from a national survey that measures patient quality perceptions in a number of dimensions. The paper singled out the Overall Impression dimension as the basis for comparing ownership categories. The primary care centers were divided into five different ownership categories: Public, Independent for-profit, Independent not-for-profit, Company group for-profit, and Company group not-for-profit. All five ownership categories are funded through taxes according to specific regulations in each region.

The paper shows that Independent for-profit, as a group, gets higher scores on Overall Impression, compared to Company group, for-profit, and public centers. The number of not-for-profit centers is small and it is hard to draw any conclusions for them. Based on the small number of not-for-profit centers in the sample, however, they get very good scores. Based on the findings reported in this paper, we have contributed to the discussion on perceived quality in health care and whether it differs between different ownership categories. The major contribution of the paper is to introduce the distinction between four categories of private ownership, and to analyze how perceived patient quality differs between them. Future research can potentially compare other countries, and also contribute with deeper explanations for the differences found in this paper.

## Figures and Tables

**Table 1:** Percentage of public and private primary care centers over the median for overall impression in each of the 21 regions of Sweden

Region	Percentage of *public* primary care centers with a score over median 2017 (2015 in brackets) out of total number of public centers	Number of public primary care centers with valid answers in both 2015 and 2017 surveys	Percentage of *private* primary care centers with a score over median 2017 (2015 in brackets) out of total number of private centers	Number of private primary care centers with valid answers in both 2015 and 2017 surveys
Blekinge	46 (31)	13	67 (50)	6
Dalarna	48 (44)	25	60 (80)	5
Gotland	50 (50)	4	100 (50)	2
Gävleborg	42 (34)	26	63 (69)	16
Halland	37 (33)	24	67 (67)	24
Jämtland	43 (43)	21	100 (100)	4
Jönköping	40 (50)	30	77 (46)	13
Kalmar	48 (44)	27	63 (75)	8
Kronoberg	55 (45)	20	50 (60)	10
Norrbotten	52 (48)	27	50 (75)	4
Skåne	43 (43)	83	57 (57)	65
Stockholm	36 (36)	69	58 (53)	137
Sörmland	44 (44)	18	67 (67)	9
Uppsala	29 (29)	21	75 (54)	20
Värmland	39 (43)	23	88 (71)	8
Västerbotten	47 (47)	32	80 (80)	5
Västernorrland	52 (52)	21	50 (50)	10
Västmanland	36 (36)	11	56 (56)	16
Västra Götaland	44 (44)	108	59 (59)	81
Örebro	48 (48)	25	67 (67)	3
Östergötland	50 (50)	34	44 (44)	9
TOTAL		662		455

**Table 2:** Distribution between the four categories of owners for the private providers above and below the median in each region and totally for Sweden

	Distribution between the four categories of owners for the private providers above the median in each region	Distribution between the four categories of owners for the private providers below the median in each region
Blekinge	*n* = 4	*n* = 2
	Ifp = 25%	
	Infp = 25%	
	Gfp = 50%	Gfp = 100%
Dalarna	*n* = 3	*n* = 2
	Ifp = 100%	Ifp = 50%
		Gfp = 50%
Gotland	*n* = 2	*n* = 0
	Gfp = 100%	
Gävleborg	*n* = 10	*n* = 6
	Ifp = 50%	Ifp = 33.3%
	Gfp = 50%	Gfp = 66.6%
Halland	*n* = 16	*n* = 8
	Ifp = 50%	Ifp = 12.5%
	Infp = 12.5%	
	Gfp = 37.5%	Gfp = 87.5%
Jämtland	*n* = 4	*n* = 0
	Ifp = 50%	
	Infp = 50%	
Jönköping	*n* = 10	*n* = 3
	Ifp = 70%	Ifp = 33.3%
	Gfp = 10%	Gfp = 66.6%
	Gnfp = 20%	
Kalmar	*n* = 5	*n* = 3
	Ifp = 80%	Ifp = 100%
	Gfp = 20%	
Kronoberg	*n* = 5	*n* = 5
	Ifp = 40%	
	Infp = 40%	Infp = 20%
	Gfp = 20%	Gfp = 80%
Norrbotten	*n* = 2	*n* = 2
		Ifp = 50%
	Gfp = 100%	Gfp = 50%
Skåne	*n* = 38	*n* = 27
	Ifp = 21%	Ifp = 25.9%
	Gfp = 79%	Gfp = 74.1%
Stockholm	*n* = 79	*n* = 58
	Ifp = 44.3%	Ifp = 24.1%
	Infp = 3.8%	Infp = 1.7%
	Gfp = 50.6%	Gfp = 74.2%
	Gnfp = 1.3%	
Sörmland	*n* = 6	*n* = 3
	Ifp = 16.6%	Ifp = 100%
	Gfp = 83.3%	
Uppsala	*n* = 15	*n* = 5
	Ifp = 40%	
	Gfp = 60%	Gfp = 100%
Värmland	*n* = 7	*n* = 1
	Ifp = 14.3%	
	Gfp = 85.7%	Gfp = 100%
Västerbotten	*n* = 4	*n* = 1
	Ifp = 50%	
	Gfp = 50%	Gfp = 100%
Västernorrland	*n* = 5	*n* = 5
	Ifp = 20%	Ifp = 40%
	Infp = 40%	
	Gfp = 20%	Gfp = 60%
Västmanland	*n* = 9	*n* = 7
	Ifp = 33.3%	Ifp = 28.5%
	Gfp = 66.6%	Gfp 71.5%
Västra Götaland	*n* = 48	*n* = 33
	Ifp = 37.5%	Ifp = 24.2%
	Infp = 2.1%	
	Gfp = 58.3%	Gfp = 69.7%
	Gnfp = 2.1%	Gnfp = 6.1%
Örebro	*n* = 2	*n* = 1
	Ifp = 50%	
	Gfp = 50%	Gfp = 100%
Östergötland	*n* = 4	*n* = 5
	Ifp = 25%	Ifp = 20%
	Gfp = 75%	Gfp = 80%
Total Sweden	*n* = 278	*n* = 177
	Ifp = 39.2% ( *n* = 109)	Ifp = 26% ( *n* = 46)
	Infp = 4.7% ( *n* = 13)	Infp = 1.1% ( *n* = 2)
	Gfp = 54.7% ( *n* = 152)	Gfp = 71.8% ( *n* = 127)
	Gnfp = 1.4% ( *n* = 4)	Gnfp = 1.1% ( *n* = 2)

**Table 3:** Comparison of patient quality perceptions between public care centers and the four types of private care centers, for each region and for Sweden as a whole

	Independent for-profit ( *n* = 155)	Independent not-for-profit ( *n* = 15)	Group for-profit ( *n* = 279)	Group not-for-profit ( *n* = 6)	Public ( *n* = 662)
Blekinge	1 above median	1 above median	2 above median		6 above median
			2 below median		7 below median
Dalarna	3 above median				12 above median
	1 below median		1 below median		13 below median
Gotland			2 above median		2 above median
					2 below median
Gävleborg	5 above median		5 above median		11 above median
	2 below median		4 below median		15 below median
Halland	8 above median	2 above median	6 above median		9 above median
	1 below median		7 below median		15 below median
Jämtland	2 above median	2 above median			9 above median
					12 below median
Jönköping	7 above median		1 above median	2 above median	12 above median
	1 below median		2 below median		18 below median
Kalmar	4 above median		1 above median		13 above median
	3 below median				14 below median
Kronoberg	2 above median	2 above median	1 above median		11 above median
		1 below median	4 below median		9 below median
Norrbotten	1 below median		2 above median		14 above median
			1 below median		13 below median
Skåne	8 above median		30 above median		37 above median
	7 below median		20 below median		46 below median
Stockholm	35 above median	3 above median	40 above median	1 above	24 above median
	14 below median	1 below median	43 below median		45 below median
Sörmland	1 above median		5 above median		8 above median
	3 below median				10 below median
Uppsala	6 above median		9 above median		6 above median
			5 below median		15 below median
Värmland	1 above median		6 above median		9 above median
			1 below median		14 below median
Västerbotten	2 above median		2 above median		15 above median
			1 below median		17 below median
Västernorrland	1 above median	2 above median	2 above median		11 above median
	2 below median		3 below median		10 below median
Västmanland	3 above median		6 above median		4 above median
	2 below median		5 below median		7 below median
Västra Götaland	18 above median	1 above median	28 above median	1 above median	48 above median
	8 below median		23 below median	2 below median	60 below median
Örebro	1 above median		1 above median		12 above median
			1 below median		13 below median
Östergötland	1 above median		3 above median		17 above median
	1 below median		4 below median		17 below median
Summary Sweden total number above median and total number below median	109 above	13 above	152 above	4 above	290 above
	46 below	2 below	127 below	2 below	372 below
